# Hybrid Drug Delivery Patches Based on Spherical Cellulose Nanocrystals and Colloid Titania—Synthesis and Antibacterial Properties

**DOI:** 10.3390/nano8040228

**Published:** 2018-04-08

**Authors:** Olga L. Evdokimova, Fredric G. Svensson, Alexander V. Agafonov, Sebastian Håkansson, Gulaim A. Seisenbaeva, Vadim G. Kessler

**Affiliations:** 1 G.A. Krestov Institute of Solution Chemistry of the Russian Academy of Sciences, Akademicheskaya St.1, 153045 Ivanovo, Russia; olga_evdokimova@outlook.com (O.L.E.); ava@isc-ras.ru (A.V.A.); 2 Department of Molecular Sciences, BioCenter, Swedish University of Agricultural Science, 750 07 Uppsala, Sweden; fredric.svensson@slu.se (F.G.S.); sebastian.hakansson@slu.se (S.H.); gulaim.seisenbaeva@slu.se (G.A.S.)

**Keywords:** titania, cellulose nanocrystals, drug delivery, bioactivity, triclosan

## Abstract

Spherical cellulose nanocrystal-based hybrids grafted with titania nanoparticles were successfully produced for topical drug delivery. The conventional analytical filter paper was used as a precursor material for cellulose nanocrystals (CNC) production. Cellulose nanocrystals were extracted via a simple and quick two-step process based on first the complexation with Cu(II) solution in aqueous ammonia followed by acid hydrolysis with diluted H_2_SO_4_. Triclosan was selected as a model drug for complexation with titania and further introduction into the nanocellulose based composite. Obtained materials were characterized by a broad variety of microscopic, spectroscopic, and thermal analysis methods. The drug release studies showed long-term release profiles of triclosan from the titania based nanocomposite that agreed with Higuchi model. The bacterial susceptibility tests demonstrated that released triclosan retained its antibacterial activity against *Escherichia coli* and *Staphylococcus aureus*. It was found that a small amount of titania significantly improved the antibacterial activity of obtained nanocomposites, even without immobilization of model drug. Thus, the developed hybrid patches are highly promising candidates for potential application as antibacterial agents.

## 1. Introduction

Antibiotic resistance of bacteria and other microorganisms is a serious public health concern and a major cause of morbidity and mortality worldwide [1,2]. It is well known that the spread of the bacteria resistance against a variety of antibiotics is due to excessive use of drugs, as well as the misapplication of medicines and inappropriate prophylaxis [3]. At present, it is an urgent necessity to prevent the spread the antimicrobial resistance and to limit the unnecessary use of antibiotics [4].

Nowadays, numerous studies are concentrated on finding efficient pathways to produce new types of highly efficient and low-cost antibacterial agents [5]. Among them, hybrid organic–inorganic nanomaterials are attracted considerable interest in the field of pharmaceutical and biomedical applications [6,7]. Due to synergistic combination of the unique properties of inorganic nanoparticles with chemical features derived from the morphology and the microstructure of polymers, these hybrid materials turned out to be promising candidates for potential application in nanomedicine, advanced diagnostics for cell targeting [8] and imaging, drug delivery, tissue engineering technologies and nano-containers [9,10], nano-reactors, optics [11], biosensors [12], catalysts [13], absorbents [14]. Nanocellulose, as a renewable natural biopolymer, has been explored as a novel nanostructured material for drug administration and its controlled delivery [15], for immobilization and recognition of enzyme/protein, and as skin and bone tissue repair materials, tissue bioscaffolds for cellular culture [16]. The excellent physical properties, special surface chemistry, and biological properties (biocompatibility, biodegradability, and low toxicity) of nanocellulose allow it to be used as an excipient or biomatrix for loading and delivery of diagnostic or therapeutic agents and different types of drugs [15,17]. As an inorganic component, nanosized TiO_2_ has gained much attention from both theoretical and practical point of view as a biocompatible nanomaterial with outstanding properties for bioencapsulation and drug delivery [18,19], and as container or carrier for delivery of small molecule and macromolecular drugs [20]. However, a serious drawback of such systems is uncontrollable and burst release of drugs, which can lead to toxic levels. 

The increasing antibiotic resistance of bacteria in the treatment of wounds has led to the renaissance of transdermal/topical drug delivery for the treatment of wound infections by local application of antiseptic drugs [21,22]. Compared to the conventional oral/parenteral delivery routes, drug delivery through skin provides controlled and constant administration of drugs, and offers an enhancement of local concentration of the drugs without the necessity of frequent dressing changes [23]. For example, R. Kolakovic et al. [24] reported the application of nanofibrillar cellulose as a matrix-forming material for long-lasting sustained delivery of indometacin. An active wound dressing based on bacterial nanocellulose loaded with the antiseptic octenidine was developed as drug delivery system for the treatment of acute and chronically infected wounds [25]. In another work, new controlled-release carriers based on bacterial nanocellulose were developed for berberine hydrochloride delivery [26]. Thus, the development of nanocellulose based composites for transdermal drug delivery is an actively developing direction. The studies in this field were performed only for very limited classes of drugs, and now required further attention. Although research on nanocomposites based on nanocellulose and inorganic nanoparticles is exponentially growing, there are a few reports that have been published on the synthesis of titania–nanocellulose composites for drug delivery [27,28,29,30]. Inspired by our previous work [29,30], we continued our investigation in the field of developing transdermal drug delivery systems. This time, we present a simple approach to develop a novel type of nanocomposites based on spherical-shaped cellulose nanocrystals and titania nanoparticles with chemical grafting of the model drug, with the final objective of obtaining a highly efficient transdermal drug delivery system. For the first time, the conventional filter paper was applied as a model source for direct production of cellulose nanocrystals with the spherical-shaped morphology. Triclosan (2,4,4′-trichloro-2′-hydroxydiphenyl ether) was chosen as a non-ionic, broad-spectrum antibacterial and antifungal model drug approved by a Food and Drug Administration (FDA) [31]. 

## 2. Materials and Methods

### 2.1. Materials

Copper sulfate (CuSO_4_·5H_2_O), sodium hydroxide (NaOH), ammonia (NH_4_OH, 25 wt %), sulfuric acid (H_2_SO_4_, 98 wt %), 1,2,3,4-bytanetetracarboxylic acid (BTCA, [-CH(CO_2_H)CH_2_CO_2_H]_2_ M_w_ 234.18), sodium hypophosphite (NaH_2_PO_2_, M_w_ 87.98), triclosan (Irgasan, C_12_H_7_Cl_3_O_2_, M_w_ 289.54) were purchased from Sigma-Aldrich Sweden AB (Stockholm, Sweden) and used without further purification. The TiO_2_ nanosol was produced by CaptiGel AB, Uppsala, Sweden. Munkllerfilter paper (100% cotton linters with an ash content of 0.007%) was used as a precursor material for cellulose nanocrystals (CNC) production.

### 2.2. Synthesis of Spherical Cellulose Nanocrystals

**Scheme 1:**
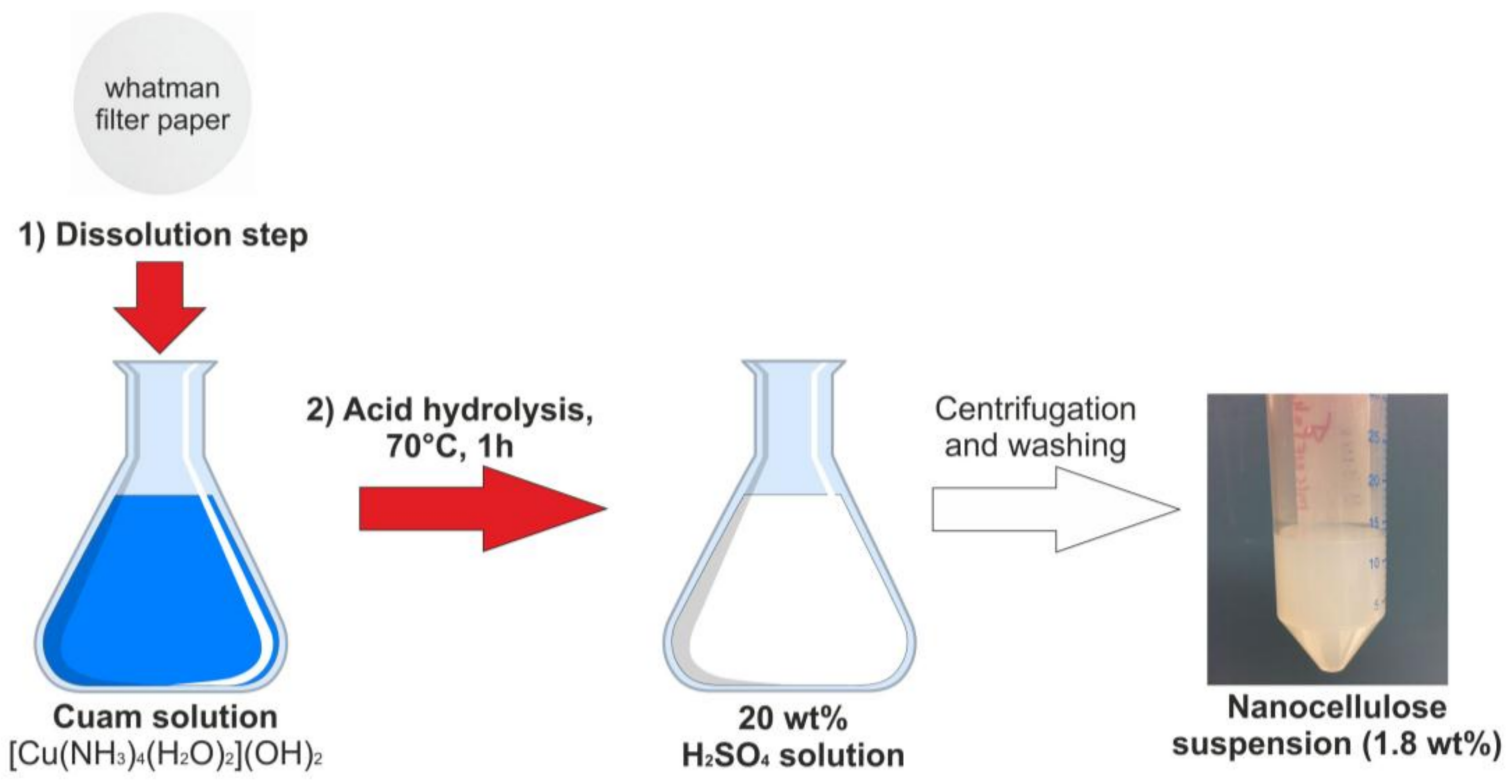
Synthesis route of spherical shaped nanocellulose from filter paper (PCNC sample).

Spherical-shaped cellulose nanocrystals (CNC) were isolated via the two-step process involving initial dissolution of the filter paper into tetraamminediaquacopper dihydroxide (cuam, Schweitzer’s reagent) with further regeneration by acid hydrolysis with a 20 wt % sulfuric acid. No chemical pretreatment procedures of the filter paper have been done. The synthesis route is illustrated in Scheme 1.

In particular, to prepare cuprammonium solution, 5 g of copper (II) sulfate was firstly dissolved in 100 mL of distilled water, and then sodium hydroxide (5 M) was added until precipitation. The copper hydroxide precipitate was thoroughly washed with distilled water to remove Na^+^. Then, the precipitate was dissolved in 200 mL of ammonia (25 wt %) giving a deep blue solution of cuam. Then, the desired amount of the filter paper was completely dissolved in the obtained solution. Next, 25 mL of Schweitzer’s reagent solution, containing the dissolved filter paper, were added into 100 mL of 20 wt % of the sulfuric acid solution and stirred vigorously at 70 °C for 1 h. After that, hydrolysis was immediately quenched by adding 500 mL of cold water to the reaction mixture. The resulting nanocellulose slurry was separated from the sulfuric acid by several cycles of centrifuging and washing with distilled water until pH = 6 and stored at 4 °C before further use.

### 2.3. Bionanocomposite Films Preparation

**Scheme 2:**
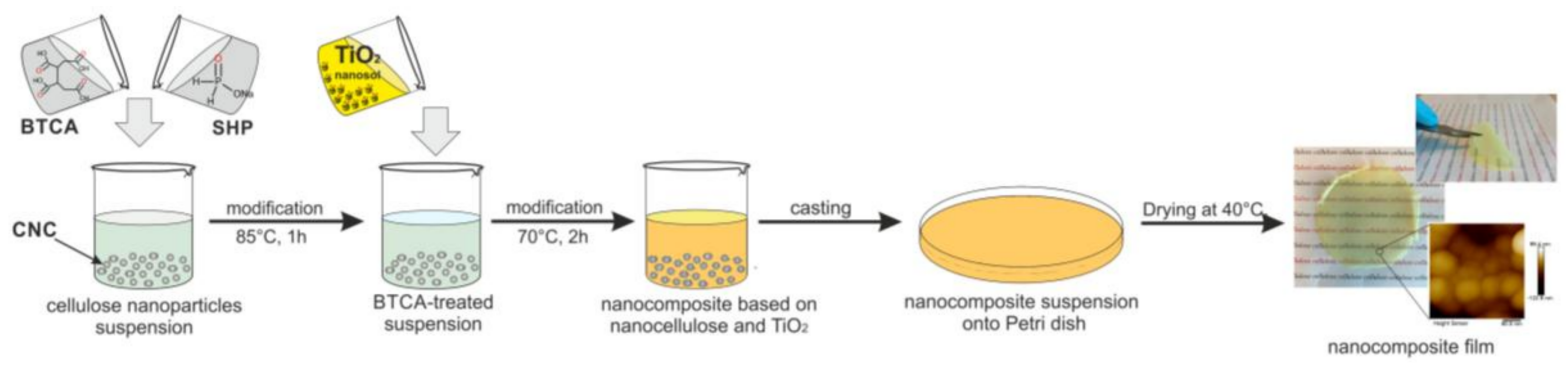
The preparation of dried nanocomposite film based on nanocellulose and titania nanocrystals (CNC_TiO_2_ sample).

Firstly, to crosslink titania nanoparticles with cellulose nanocrystals, 1,2,3,4-butanetetracarboxylic acid (BTCA) was used as a spacer in the presence of sodium hypophosphite (SHP) as a catalyst. The nanocellulose slurry (5.5 g, 1.8 wt %) obtained from the filter paper was treated by BTCA (6.8 × 10^−4^ mol) with SHP (50% by weight of BTCA) aqueous solution at 85 °C during 1 h. To obtain nanocomposite based on cellulose nanocrystals and titania nanoparticles, titania nanosol was added to an aqueous suspension of the BTCA-treated nanocellulose and kept at 70 °C for 2 h. Nanocellulose based nanocomposite grafted with titania and triclosan was prepared by the following procedure (Scheme 2).

Drug grafting was performed in amounts calculated in the assumption of the formation of a uniform, single layer coverage on TiO_2_-modified cellulose nanocrystals (see ESI for explanation). For this purpose, 4.74 × 10^−5^ mol of triclosan powder was dissolved in 1 mL of ethanol and then added to titania nanosol (1.5 × 10^−4^ mol). The obtained solution was mixed with an aqueous suspension of BTCA-treated nanocellulose and kept at 70 °C for 2 h. The final amount of TiO_2_ in the obtained nanocomposites was 3.4%. To compare, triclosan loaded nanocomposite was also synthesized without using titania as a binding agent (CNC_TR). In this case, nanocellulose slurry was mixed with TR (4.74 × 10^−5^ mol) initially dissolved in 1 mL of ethanol and kept at 70 °C during 2 h. Finally, all obtained nanocomposites were dried at 40 °C for 48 h without addition any other polymers and plasticizers. The total amount of TR in the obtained nanocomposites was 3.8 wt %, introduced as the weighted amount of solid drug.

### 2.4. Characterization

Atomic force microscope (AFM) was used to analyze dimensions and compare the surface morphology of the obtained nanomaterials. For this purpose, the AFM measurements were performed by using a Bruker Dimension FastScan Atomic Force Microscope. Image analysis was performed using the in-built particle analysis option of NanoScope Analysis 1.7 software (Bruker, Billerica, MA, USA), which generates histograms of particle size distribution. Scanning Electron microscopy (SEM) images of the samples were obtained by a Hitachi TM-1000 scanning electron microscopy (Spectral Solutions AB, Sweden). IR spectra of the freeze-dried samples were obtained with a Perkin Elmer FT-IR spectrometer Spectrum-100 (Blue Scientific, Cambridge, UK). A total of 8 or 16 scans were carried out between 400 cm^−1^ and 4000 cm^−1^ in transmittance mode. All spectra were smoothed and baseline corrected. Thermo-gravimetric analysis was carried out in air at a heating rate of 10 °C/min, using a Perkin-Elmer TGA-7 or Pyris 1 device. The X-ray powder diffraction (XRD) studies were carried out at room temperature using a Bruker APEX II CCD diffractometer (Mo Kα 0.71, graphite-monochromator).

The tensile properties of the obtained nanocellulose films were measured by testing machine 2099-P-5 (“Tochpribor”, Novosibirsk, Russia) at a crosshead speed of 0.5 mm min^−1^ at room temperature. Prior to analysis, the samples were cut a width of 15 mm and a length of 30 mm. The film thickness was measured by a micrometer. For this purpose, the thicknesses taken from six random positions on the film was detected, and the average values were used in the calculation. Three measurements were performed for each sample, and the average values were calculated. The Young’s modulus (elastic modulus) was calculated from the slope of the initial linear section on the stress–strain curve [32].

### 2.5. In Vitro Drug Release

To investigate the release profile of TR, the nanocomposite films containing TiO_2_ and TR were incubated in 300 mL of acetate buffer solution with addition of 5 vol % of ethanol (0.2 M, pH = 5.5 as natural skin surface pH) at constant temperature (37 ± 0.5 °C) on constant stirring at 100 rpm [33]. The addition of small amounts of ethanol to the release medium was proposed in [33] to slightly enhance the solubility of triclosan and facilitate its determination by UV–vis spectrophotometry. The calibration curve for the determination of TR in the obtained buffer was linear (R^2^ = 0.99, range of 5–50 mg/mL). At determined time intervals, 1 mL of each solution was taken out for analysis, and the same volume of fresh medium was added to maintain a constant volume. TR content in each aliquot was determined spectrophotometrically at 279 nm against a blank solution. UV–vis quantitative analysis of the released drug was performed on a UV/vis spectrophotometer UV-1800 (Shimadzu, Kyoto, Japan). A linear calibration curve for TR was obtained at 279 nm. The released drug was determined by using the following equation:

Cumulative drug release (%) = (released drug)/(loaded drug) × 100,

where the released drug was calculated from the drug concentration measured in the total volume and the total drug was the amount loaded in the obtained sample. The loaded amount of triclosan was determined by the following procedure. The obtained suspension modified by crosslinking agent (BTCA) and triclosan mixed with TiO_2_ nanosol (please, see Section 2.3) was centrifuged and the supernatant was analyzed for triclosan in acetate buffer solution with addition of 5 vol % of ethanol (0.2 M, pH = 5.5 as natural skin surface pH) at constant temperature (37 ± 0.5 °C) on constant stirring at 100 rpm) at 279 nm by using spectrophotometer (UV/vis spectrophotometer UV-1800, Shimadzu, Kyoto, Japan):

Loaded TR (%) = (total drug − free drug)/(total drug) × 100

The cumulative amounts of drug released from the obtained nanocomposites were plotted against time.

### 2.6. Mathematical Modelling of Release Kinetics

To examine the drug release kinetics, the in vitro drug release data was fitted to various release kinetic models using the following equations [34,35]:

(1) $$\text{Zero-order model: }Q_{t}=Q_{o}-k_{o}t$$

(2) $$\text{First-order model: }\ln Q_{t}=\;\ln Q_{o}-k_{1}t$$

where $Q_{t}$ is the amount of drug released at time *t*, $Q_{o}$ is the initial amount of drug in solution, $k_{o}$ and $k_{1}$ is the zero-order and the first-order release constant, respectively;

(3) $$\text{Higuchi model: }Q_{t}=k_{H}\sqrt{t}$$

where $Q_{t}$ is the amount of drug released in time *t*, $k_{H}$ is the release rate constant for the Higuchi model;

(4) Hixson–Crowell cube root model: (Wo)13−(Wt)13=kHCt

where $W_{o}$ is the initial amount of the drug in the film, $W_{t}$ is the amount of drug released in time *t*, and $k_{HC}$ is the rate constant for Hixson–Crowell rate equation;

(5) Korsemeyer–Peppas model: MtM∞=kKPtn

where MtM∞ is the fraction of drug released and $k_{KP}$ is a constant characteristic of the drug–polymer system, and *n* is the diffusional/release exponent.

### 2.7. In Vitro Antibacterial Studies 

The disk diffusion method (EUCAST, 2014) was used to assay the antibacterial activity of the obtained nanocomposite against test strains *S. aureus* CCUG1800T and *E. coli* CCUG24T on MH agar plates. An inoculum of the test organism was swabbed onto the surface of the agar plate, PCNC, CNC_TiO_2_, and CNC_TiO_2__TR samples were placed on the agar. The plates were incubated for 18 h at 37 °C, and the clear zones around the antibacterial agents were then measured. The minimum inhibitory concentration (MIC) of TR has been used as 0.1 mg/mL in accordance with Escalada et al. [36]. Experiments were performed in triplicates.

### 2.8. Molecular Model Compounds

*[Ti_4_(µ_3_-O)_2_(µ_2_-OEt)_2_(C_9_H_16_O_3_)_2_(C_8_H_12_O_3_)_2_(C_12_H_6_Cl_3_O_2_)_2_·4C_3_H_6_O]*, (**1**). To 0.135 g triclosan (0.46 mmol) in anhydrous acetone, 0.30 mL (1.43 mmol) titanium(IV) ethoxide was added under nitrogen atmosphere. This resulted in a bright yellow clear solution. After heating to ~40 °C, the reaction mixture was stored at −18 °C. After ca. 6 weeks, large brown-orange crystals were obtained in nearly quantitative yield. The mother-solution had also turned orange. IR, cm^−1^: 3397 w, 1714 sh, 1583 s, 1590 s, 894 s, 816 s, 805 s. NMR ^1^H *δ* ppm: 7.54 d (*J* = 2.15 Hz) 7.28 dd (*J* = 8.94, 2.22 Hz), 7.04 d (*J* = 2.09 Hz), 6.96 d (*J* = 8.49 Hz), 6.88 dd (*J* = 8.70, 2.16 Hz), 6.83 d (*J* = 8.70 Hz), s 6.17, 3.56 q (*J* = 7.00, 6.89 Hz), 2.18 s, 1.18 s. Single-crystal X-ray diffraction data were recorded with a Bruker D8 SMART APEX II CCD diffractometer (graphite monochromator). Data for C6_2_H_78_O_20_Cl_6_Ti_4_·4(C_3_H_6_O): triclinic, P-1, a = 11.985(12), b = 13.177(11), c = 16.930(19) Å, α = 107.40(3), β = 94.90(2), γ = 115.921(12)°, V = 2221(4) Å^3^. D_calcd_ = 1.313 g/cm^3^ for Z = 1, λ(Mo-Kα) = 0.71073 Å. A total of 7040 (R_int_ = 0.0851) independent reflections were collected at 296 K up to 2θ_max_ = 50.50° (completeness = 97.6%). The structure was solved by direct methods.

*Ti_5_(µ_3_-O)_2_(µ_2_-OEt)_5_(µ-OEt)_8_(C_9_H_16_O_3_)(C_12_H_6_Cl_3_O_2_)*, (**2**). Under nitrogen atmosphere, titanium(IV) ethoxide, 50 µL (0.24 mmol) was added to 20.6 mg (0.071 mmol, 0.3 eq.) triclosan in 0.40 mL anhydrous acetone. A bright yellow clear solution was obtained. The reaction mixture was heated gently to ~40 °C and subsequently stored in freezer. Small bright yellow crystals of nearly quantitative yield were obtained after some weeks. IR, cm^−1^, 1712 m, 1582 s, 1593 m, 1488 m, 894, 815 s. NMR ^1^H *δ* ppm: 7.47 s, 7.20 d (*J* = 7.0 Hz), 7.11 s, 3.78 singlet, 7.11 singlet, 6.06 d (*J* = 8.5 Hz), 4.28 q (7.79, 6.78 Hz), 4.07 s, 3.78 s, 2.62 s, 2.61 s, 2.51 d (*J* = 12.53 Hz), 2.20 s, 2.16 s, 1.93 s, 1.92 s, 1.26 triplet (*J* = 7.2 Hz), 1.23 d (*J* = 6.11 Hz). 

Single-crystal X-ray diffraction data were recorded with a Bruker D8 SMART APEX II CCD diffractometer (graphite monochromator). Data for C_47_H_86_O_20_Cl_3_Ti_5_, triclinic P-1, a = 11.57(2), b = 14.28(3), c = 21.37(4), α = 79.83(3)°, β = 88.00(3)°, γ = 70.90(3)°. λ(Mo-Kα) = 0.71073 Å. V = 3283(10) Å-3. D_calc_ = 1.331 g/cm^3^ for Z = 2. A total of 7732 (R_int_ =0.0798) independent reflections were collected at 153 K up to 2θ_max_ = 42.5° (completeness = 96.4%)

The structure was solved by direct methods. Data for the structure is based on three data series as the crystals were highly sensitive and degraded under data collection even at low-temperature.

The details of structure investigation of compounds **1** and **2** are available free-of-charge from the Cambridge Crystallographic data base citing registration numbers **CCDC 1534781** and **1832990** respectively using the link http://www.ccdc.cam.ac.uk.

## 3. Results and Discussion

### 3.1. Preparation and Characterization of Nanocellulose Based-Nanocomposites 

**Figure 1:**
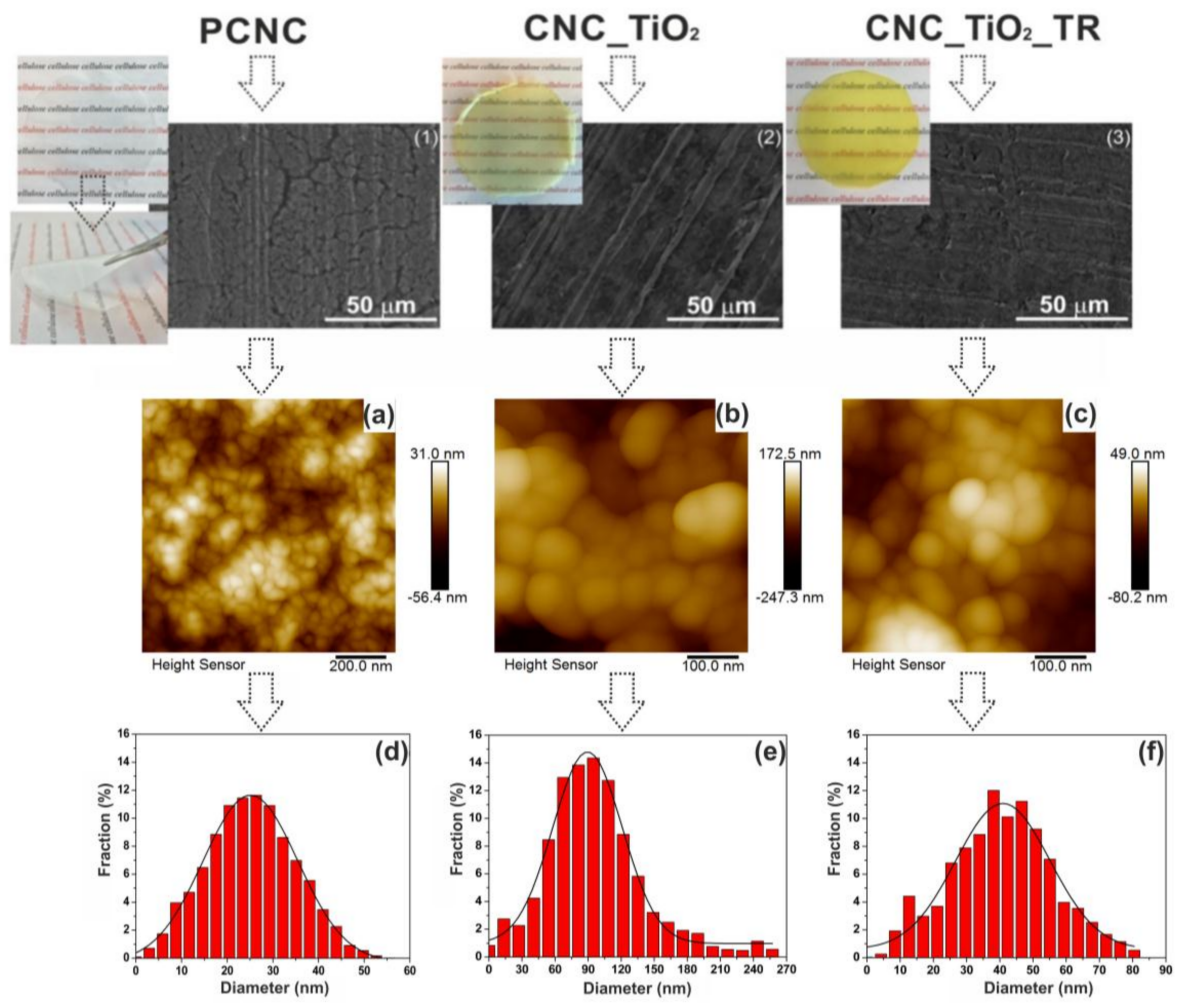
Morphology images obtained by AFM (**a**–**c**) and SEM (**1**–**3**) microscopy together with visual images, particle size distribution (theoretically fitted using Gaussian distribution function) (**d**–**f**) of the obtained samples.

At present, one of the most widely used extraction process of cellulose nanocrystals (CNC), nanofibers (CNF) or nanowhiskers (CNW) is acid hydrolysis with 63–65 wt % sulfuric acid concentration, temperature in the range of 40–60 °C, and reaction time of 1–4 h [37,38]. However, the reduction of the acid concentration to a low level is crucial due to the ecological, environmental, and also economic reasons. In this study, for the production of spherical-shaped cellulose nanocrystals, a simple and quick two-step process based on the complexation first with Cu(II) solution in aqueous ammonia, followed by acid hydrolysis with lower concentration sulfuric acid (20 wt %), was developed. For the first time, the conventional analytical filter paper was applied as a model source for direct production of spherical-shaped cellulose nanocrystals. Previously, it was reported that cellulose nanospheres could be obtained from microcrystalline cellulose by controlled hydrolysis using anaerobic microbial consortium [39], by acid hydrolysis with a mixed HCl–H_2_SO_4_ solution at 80 °C in a sonicator for 6 h [40], by the treatment with a high concentrated mixture of nitric acid (68% *w*/*w*) and hydrochloric acid (37% *w*/*w*) solutions using waste cotton fabrics as starting materials [41]. The nanocellulose-based nanocomposite film formation occurs at 40 °C by slow water evaporation without addition of any other polymers and plasticizers. Visual images of the neat nanocellulose film (PCNC), nanocomposite based on nanocellulose and TiO_2_ (CNC_TiO_2_), and nanocomposite based on nanocellulose and TiO_2_ loaded with triclosan (CNC_TiO_2__TR), are presented in Figure 1. Visual observation of the produced nanocellulose based films showed their high optical transparency and flexibility. The surface morphology of the pure spherical cellulose nanocrystals (PCNC) and the obtained nanocomposites based on them were analyzed by scanning electron microscopy (SEM) and atomic force microscopy (AFM) (Figure 1). 

As can be seen from Figure 1 (2,3), the titania is uniformly spread in the films without formation of aggregates. The titania-containing films (2) and (3) have a smoother surface than the neat PCNC film (1). High-resolution AFM images of PCNC, CNC_TiO_2_, and CNC_TiO_2__TR samples confirmed that the obtained samples have homogeneous topography and feature particles spherical in shape (Figure 1a–c). The particle size distributions of the obtained samples together with the fitted distribution function (the Gaussian curve fit) are shown in Figure 1d–f. The number average sizes of PCNC, CNC_TiO_2_, and CNC_TiO_2__TR were found to be around 25.1 ± 0.5 nm, 89.4 ± 0.9 nm, and 40.9 ± 0.7 nm, respectively.

**Figure 2:**
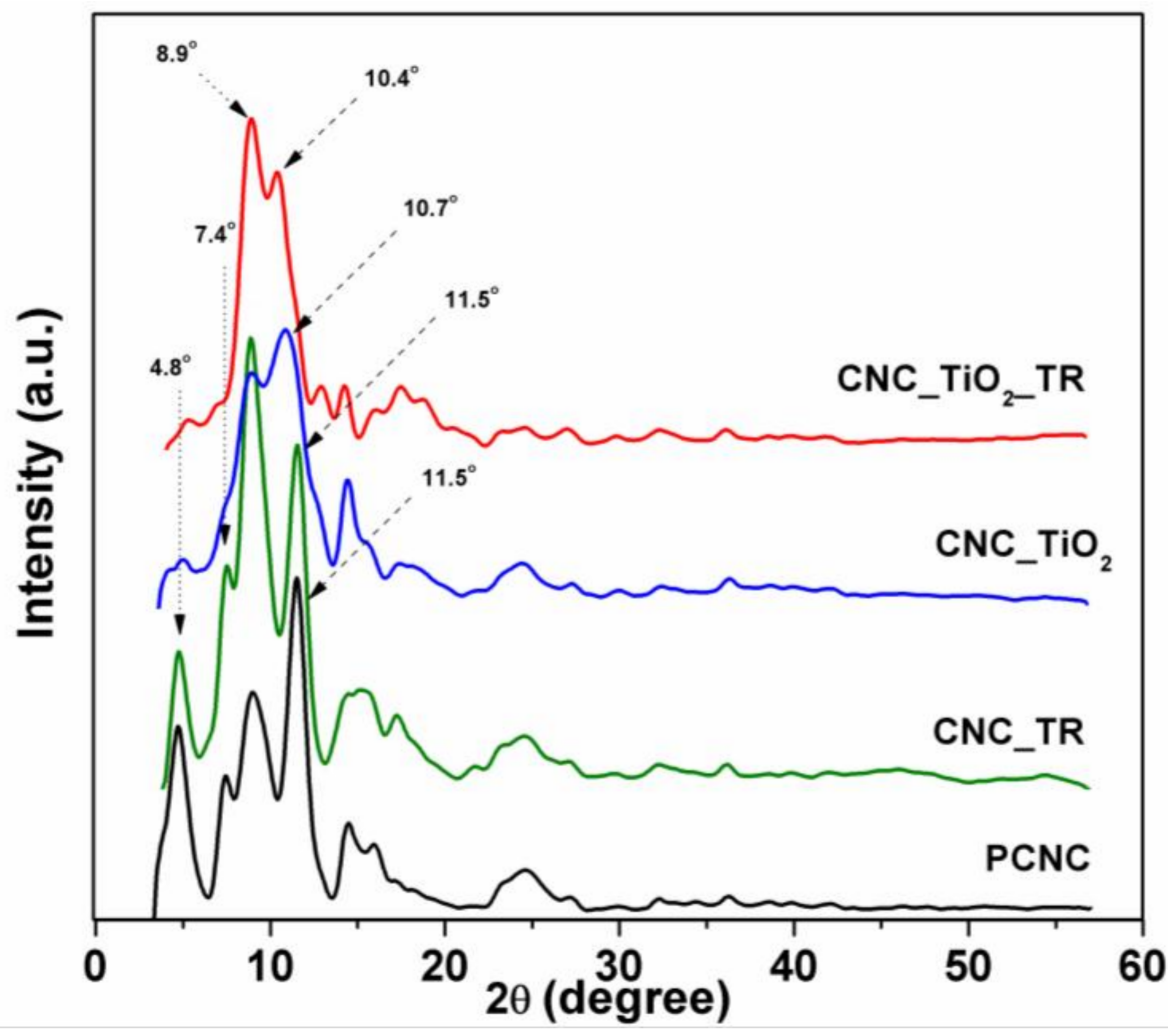
X-ray diffraction patterns of the obtained samples.

The crystal structure of the pure cellulose nanocrystals and those in nanocomposite films was determined by XRD, shown in Figure 2. All samples displayed the diffraction patterns with the presence of an amorphous broad hump and narrower peaks typical for semi-crystalline materials. It could also be noticed that all samples showed mixtures of cellulose I and II due to the appearance of the doublet in the intensity of the main peak. 

In particular, the PCNC sample exhibited a sharp doublet peak of cellulose II at 2θ = 8.9° (110) and small peak at 2θ = 16° (004) in addition to the cellulose Iβ peak at 2θ = 4.8° (001), 7.4° (110) and 11.5° (200) [42]. The CNC_TR sample showed similar diffraction pattern with the peaks at about 2θ = 4.8° (001, Iβ), 7.4° (110, Iβ), 8.9° (110, II), 11.5° (200, Iβ) and 16° (004, II). Compared to it, the main doublet peak of the CNC_TiO_2_ and CNC_TiO_2__TR samples became broader and shifted towards lower 2θ values, from 11.5° to 10.7° and 10.4°, corresponding to the (200) plane of cellulose II, respectively. Apparently, the modification of nanocellulose by titania nanoparticles has contributed to the increase of the amorphous part of the obtained materials. It is important to note that TiO_2_ has a core–shell structure with a very small (2–3 nm) crystalline anatase core and an outermost amorphous shell [43]. In this case, typical TiO_2_ diffraction peaks are difficult to detect, due to fact that the size of titania is smaller than the coherence domain required for the X-ray reflection. It is important to mention that spherically shaped cellulose nanoparticles commonly possess amorphous structure [44]. Here, XRD results clearly showed that the proposed method allows for cleavage in most of the amorphous regions, leaving behind highly crystalline cellulose.

**Figure 3:**
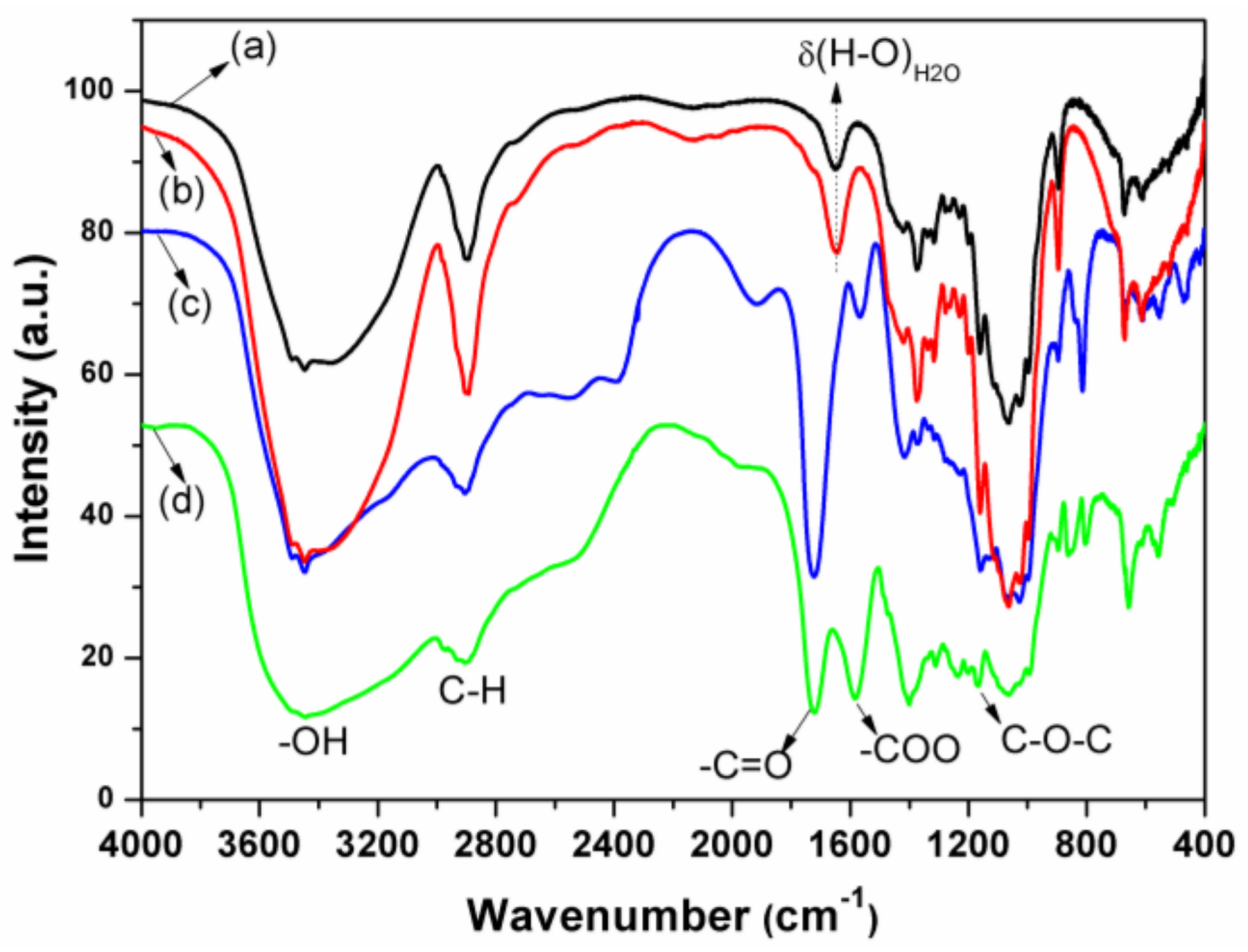
FT-IR spectra of PCNC (a), CNC_TR (b), CNC_TiO_2_ (c), and CNC_TiO_2__TR (d).

Figure 3 presents the FT-IR spectra for the pure cellulose nanocrystals (PCNC), nanocomposite based on nanocellulose and TiO_2_ (CNC_TiO_2_), and nanocomposite based on nanocellulose and TiO_2_ loaded with triclosan (CNC_TiO_2__TR). The FT-IR spectra of all the samples clearly show a broad band at 3600–3000 cm^−1^ region, the peaks at 2892 cm^−1^ and 1159 cm^−1^ which are attributed to the O–H stretching vibrations in the cellulose molecules, C–H stretching vibrations, and C–O–C asymmetric stretching vibrations, respectively [45]. The absorption peak at 1643 сm^−1^ indicates water presence due to the presence of O–H bending. Although all FTIR samples were thoroughly dried prior to analysis, it was very difficult to completely eliminate water from cellulose molecules, due to strong cellulose–water interactions [46]. No new absorbance bands were observed for the CNC_TR sample, indicating that incorporation of triclosan into nanocellulose matrix without using TiO_2_ as a spacer does not change the chemical composition of the synthesized nanocomposite (CNC_TR). To obtain nanocellulose based nanocomposites grafted with titania nanoparticles via esterification process, 1,2,3,4-butanetetracarboxylic acid was used as a linker (BTCA) and sodium hypophosphite (SHP) as a nucleophilic catalyst. The formation of ester bonds in CNC_TiO_2_ is confirmed by the appearance of the two characteristic bands at 1727 cm^–1^ and around 1580 cm^−1^, corresponding to C=O ester carbonyl stretching mode and the asymmetric carboxyl carbonyl stretching mode, respectively (Figure 3c,d) [47]. After incorporation of triclosan, CNC_TiO_2__TR has the same absorbance bands, but with a shift of COO^−^ carboxyl carbonyl stretching vibration to higher wavenumbers from 1565 cm^−1^ to 1582 cm^−1^. This observation can be assumed as the formation of the bonds between TR and the nanocomposite.

In the CNC_TiO_2_ sample, the peak at 814 cm^−1^ and a shoulder 839 cm^−1^ are attributed to the stretching vibration of Ti–O–Ti and Ti–O bonds. In CNC_TiO_2__TR, the absorption peak at 814 cm^−1^ has shifted to 805 cm^−1^ due to the coordination of triclosan to TiO_2_. The Ti–O–Ti stretching at 835 cm^−1^ in the CNC_TiO_2__TR sample is not visible, probably due to an absorption by triclosan that hides this signal. 

**Figure 4:**
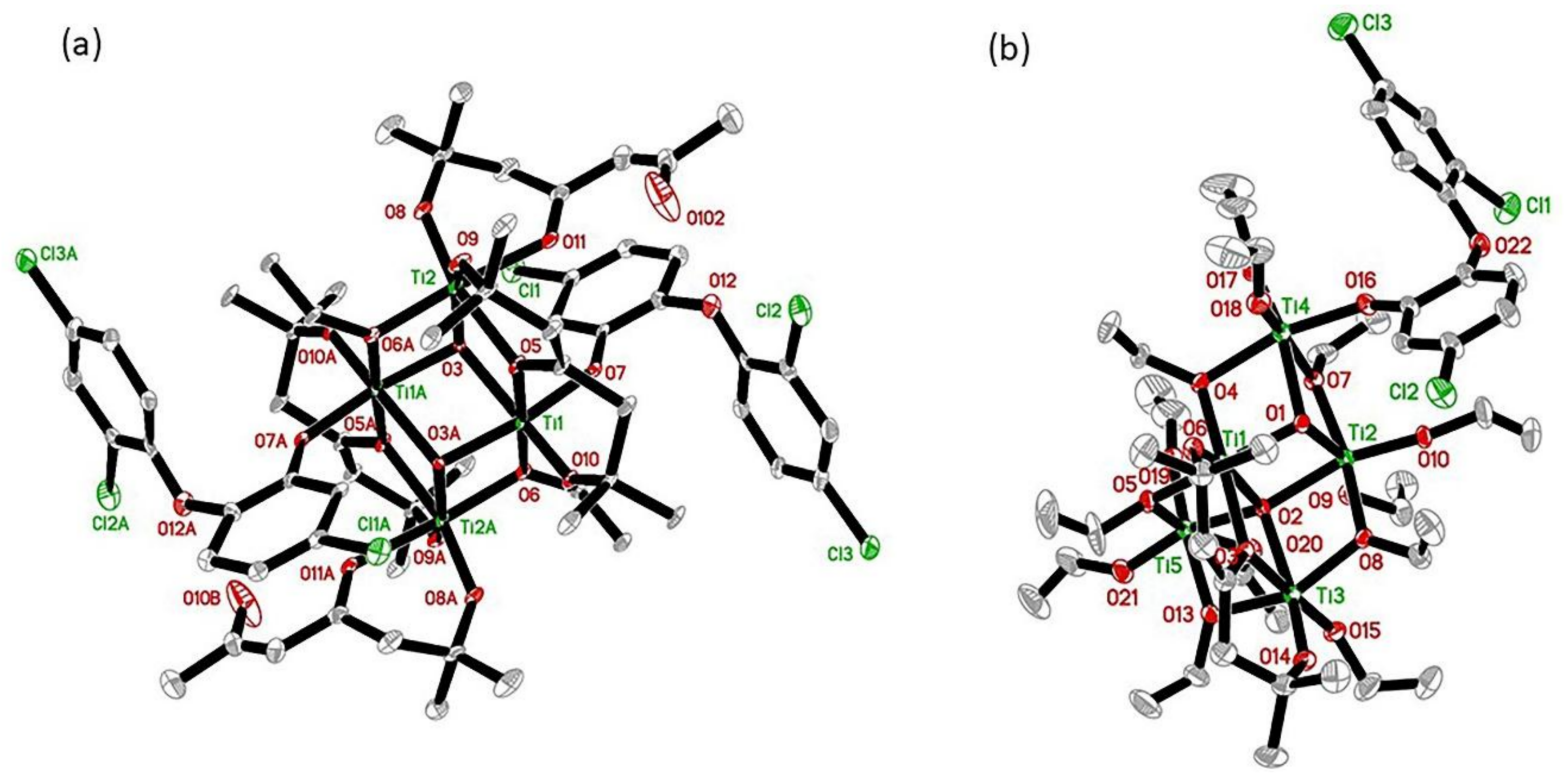
Molecular structure of compound **1** (**a**) and molecular structure of compound **2** (**b**).

To study the bonding between triclosan and titania nanoparticles, titanium oxo-complexes containing triclosan ligands were synthesized and used as models. Both compound **1** and compound **2** co-crystallized from a solution obtained by adding titanium(IV) ethoxide to a solution containing 0.3 equivalents of triclosan in anhydrous acetone after storage at −18 °C for 6 weeks. These substances resulted from alkoxide catalyzed condensation of acetone expected to produce oligonuclear model oxo-complexes. Co-crystallization of the two forms occurred because the chosen solution composition L/Ti = 0.3:1 turned intermediate between the ratios in the resulting products, which are L/Ti = 0.5:1 for compound **1** and L/Ti = 0.2:1 for compound **2**. Both species turned helpful in producing insights into ligand binding to titania surface. Isolation of pure individual compounds was not an aim of this study. Compound **1** is a triclinic tetranuclear (Ti_4_O_2_) titanium oxo-complex, belonging to the space group P-1. It contains two oxygen bridges (*μ*_3_-O) and two alkoxides bridges (*μ*_2_-O) (Figure 4a). The four titanium atoms are octahedrally coordinated and the core-structure is similar to that of anatase. Triclosan coordinates to titanium via phenoxide bonding. The aromatic rings not containing the phenol group are turned towards each other because of π–π stacking interactions. Preliminary structural characterization of the compound **1** was reported earlier in [48]. Compound **2** is a triclinic pentanuclear (Ti_5_O_2_) titanium oxo-complex (Figure 4b), also belonging to the space group P-1. The core of **2** consists of five octahedrally coordinated titanium atoms with two oxygen bridges (*μ*_3_-O), five bridging (*μ*_2_-O) ethoxide groups and eight terminal ethoxide groups. Only one triclosan ligand is attached to **2**, coordinating via phenoxide bonding just like in **1**. Also in **2**, there are π–π stacking interactions between the aromatic rings influencing packing of the molecules. 

Infrared spectra were recorded for both compound **1** and **2** in paraffin oil to avoid hydrolysis of the compounds. Vibrations for Ti–O and Ti–O–Ti (687 cm^−1^, 805 cm^−1^, and 815 cm^−1^), belonging to the titanium oxo-core were found. A signal at 894 cm^−1^ was found for both **1** and **2**, belonging to the C–O–C bond in triclosan. Medium to strong absorptions are found around 1712 cm^−1^, which are carbonyl (C=O) vibrations from the condensation products (and possibly some acetone residues). Several absorptions indicating aromatic carbons from the triclosan rings were also detected. Table S1 (Supplementary) lists some selected IR-signals. 

**Figure 5:**
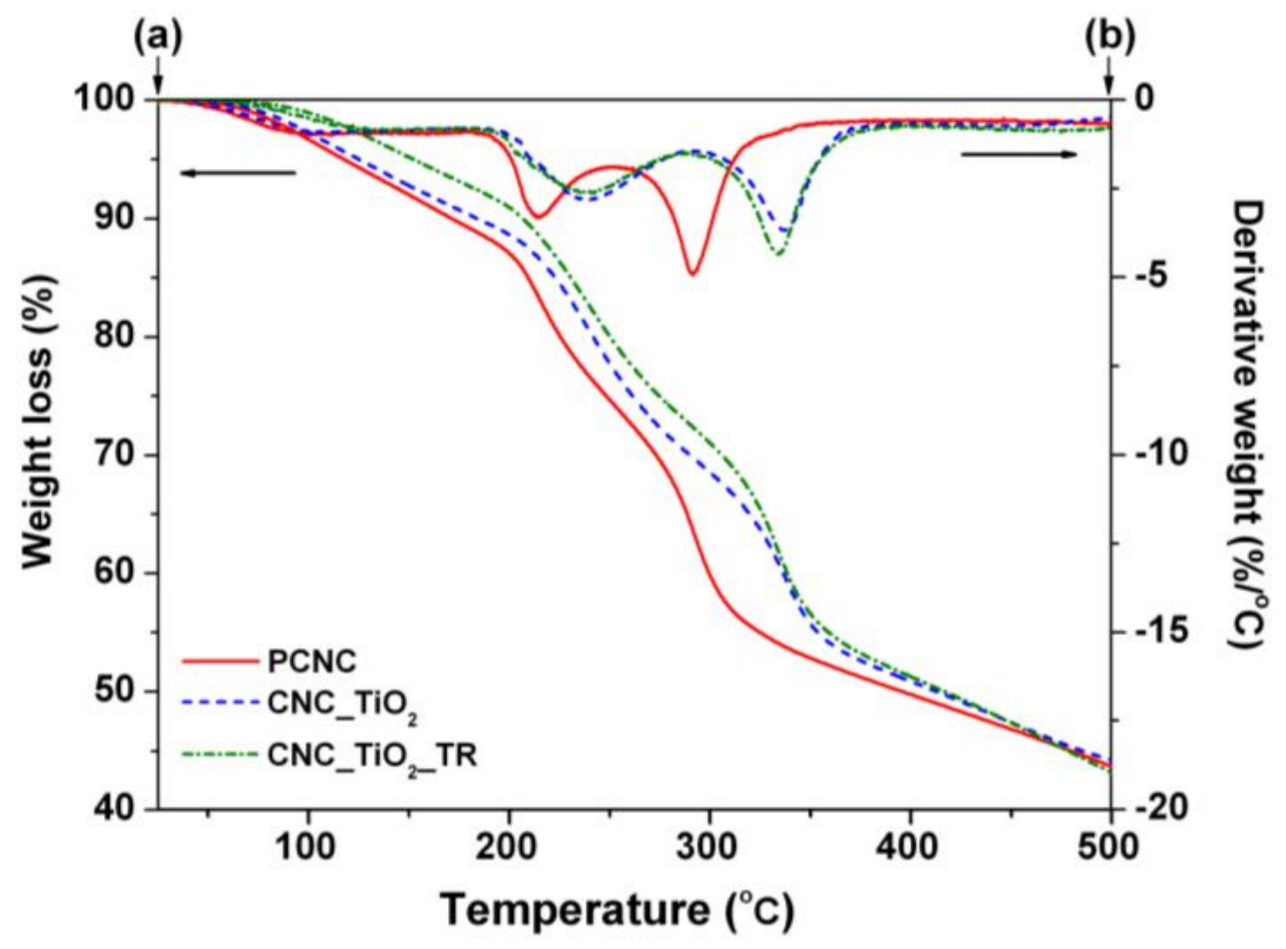
TGA (a) and DTG (b) curves of the obtained nanocomposites.

The thermal stability of the obtained nanocomposites was examined using thermogravimetric analysis. Both the TGA curves and derived curves (DTG) of the PCNC, CNC_TiO_2_, and CNC_TiO_2__TR samples have been plotted as a function of temperature and are shown in Figure 5.

It could be seen that the degradation process of all samples showed two well-separated pyrolysis processes, where one occurred between 150 °C and 250 °C, and the other between 250 °C and 500 °C. A slight weight loss was observed for all samples at around 100 °C, corresponding to the moisture evaporation [49]. The DTG plot for the PCNC sample showed the main weight loss stage at 214 °C with a maximum thermal degradation temperature at 291 °C. The first peak can be explained by the presence of unreacted carboxylic groups of BTCA that decompose at the lower temperature. A similar effect was observed by N.V. Patil and A.N. Netravali [50], who esterified mango seed starch extracted from defatted mango seed kernels using 1,2,3,4-butane-tetracarboxylic acid. Moreover, crosslinking by BTCA significantly reduced the percentage of degradation or the weight loss of the samples. The second step of thermal degradation can be attributed to the depolymerization of cellulose molecules [51]. The thermal stability of the obtained nanocomposites was higher as compared to pure cellulose nanocrystals (PCNC). For CNC_TiO_2_, the initial and maximum degradation temperature occurred at 239 °C and 337 °C, respectively. In case of CNC_TiO_2__TR, the main weight loss was obtained at 239 °C with maximum degradation temperature at 334 °C. 

**Figure 6:**
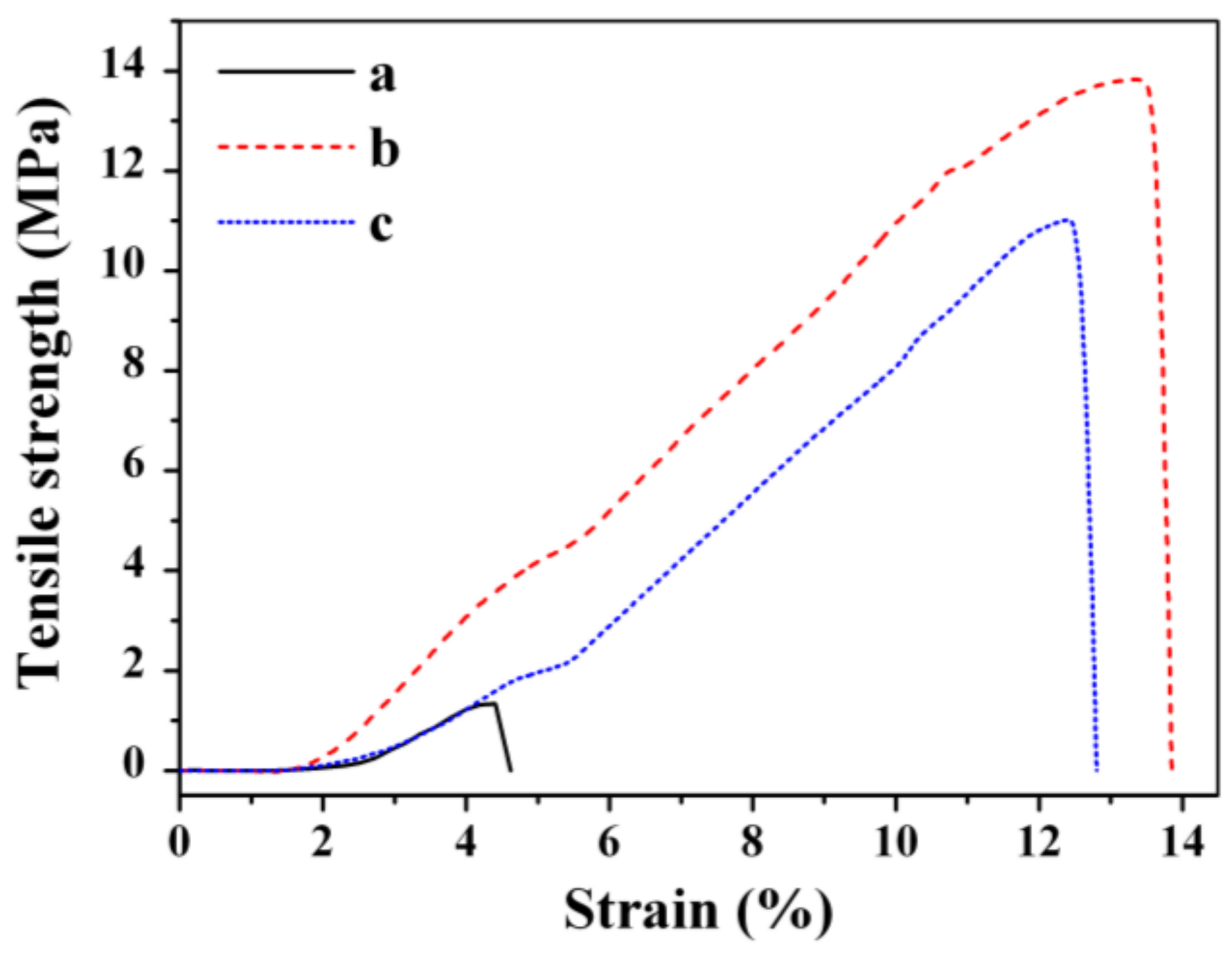
The tensile–strain curves of the films (a) PCNC, (b) CNC_TiO_2_, and (c) CNC_TiO_2__TR.

The mechanical properties of the nanocellulose based nanocomposite films were investigated by tensile testing at room temperature. The tensile–strain curves of the obtained samples are presented in Figure 6 and Table 1. It is important to mention that the films were obtained without addition of any other polymers and plasticizers. This has been reflected by the linearized behavior of the tensile–strain curves without appreciable plastic flow. The results demonstrate that modification of cellulose nanocrystals by titania nanoparticles and loading the model drug has a significant effect on the mechanical properties of the obtained films.

It was found that the tensile strength of the neat nanocellulose film (PCNC) was relatively weak (about 1.3 MPa). Compared to it, the addition of TiO_2_ enhanced the tensile strength and Young’s modulus of the nanocomposite film (CNC_TiO_2_) to 13.8 MPa and 6.8 MPa, respectively. In this case, the improved mechanical behavior of the film can be related to the reinforcing contribution of titania nanoparticles. Similar effect was observed by Schütz and co-workers [52], who studied the mechanical properties of wood-derived nanofibrillated cellulose and titania nanoparticles hybrids. The incorporation of triclosan decreased slightly the tensile strength and Young’s modulus of the nanocomposite film (CNC_TiO_2__TR) to 11.0 MPa and 6.3 MPa, respectively. One possible explanation for this is that triclosan molecules block the surface of titania nanoparticles, weakening, as a result, the interaction between TiO_2_ and cellulose. This results in poorer mechanical properties. The produced material is rather soft and is scratched even by a 2 M stift in a standard pencil test.

### 3.2. In Vitro Drug Release Studies and Kinetics

The main objective of the present study was to develop the hybrid nanostructured composites based on spherical-shaped cellulose nanocrystals and titania nanoparticles as potentially highly efficient transdermal drug delivery systems. Triclosan was chosen as a model drug because of its broad antibacterial activity against a wide range of Gram-positive and Gram-negative bacteria, as well as molds, yeasts, and parasites responsible for malaria and toxoplasmosis. Nowadays, a substantial amount of literature has been published on the application of triclosan for oral drug delivery [53]. In this work, we applied triclosan for model topical/transdermal delivery through the complexation with titania nanoparticles and further introduction into the spherical cellulose crystal-based nanocomposite. The in vitro cumulative release profiles of TR from the obtained nanocomposite (CNC_TiO_2__TR) in comparison with the CNC_TR sample obtained without using titania as a binding agent are shown in Figure 6. It is apparent that the release of the drug from the nanostructured composites depends strongly on the interaction between the drug and the biopolymer matrix.

**Figure 7:**
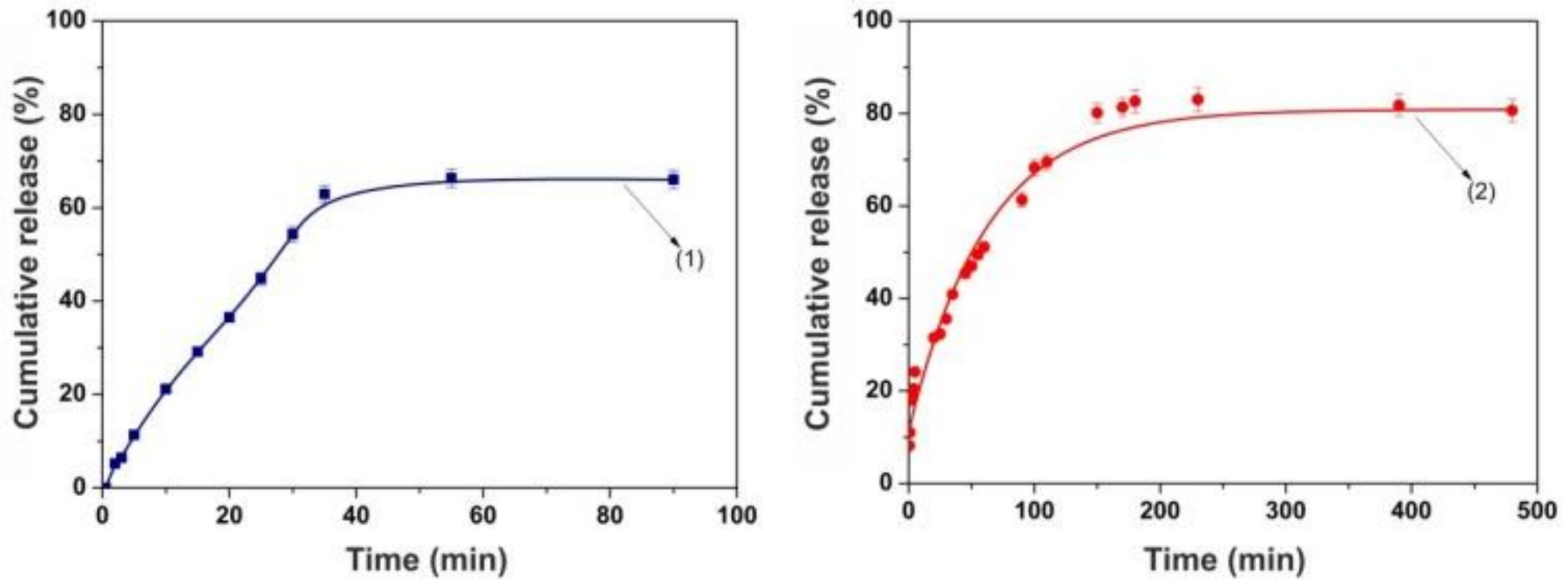
The cumulative release (%) of triclosan from CNC_TR (left) and CNC_TiO_2__TR (right).

In particular, the CNC_TR sample showed a burst release profile free-setting about ~63% of triclosan within 35 min. This may be due to physically adsorbed drug molecules on the surface of the nanocomposite. At the same time, application of the TiO_2_ exhibited a considerably slower release of triclosan from the nanocomposite CNC_TiO_2__TR. This sample displayed a sustained long-term release profile of triclosan with the rapid initial release within the first 10 min and about 83% of the drug in a controlled manner over 3.5 h (Figure 7 (right)). Such two-step release profile of the drug can be attributed to physical and chemical entrapping of the triclosan. Thus, the incorporated drug can be delivered at a constant dose transdermal from simple nanocellulose matrices. 

**Figure 8:**
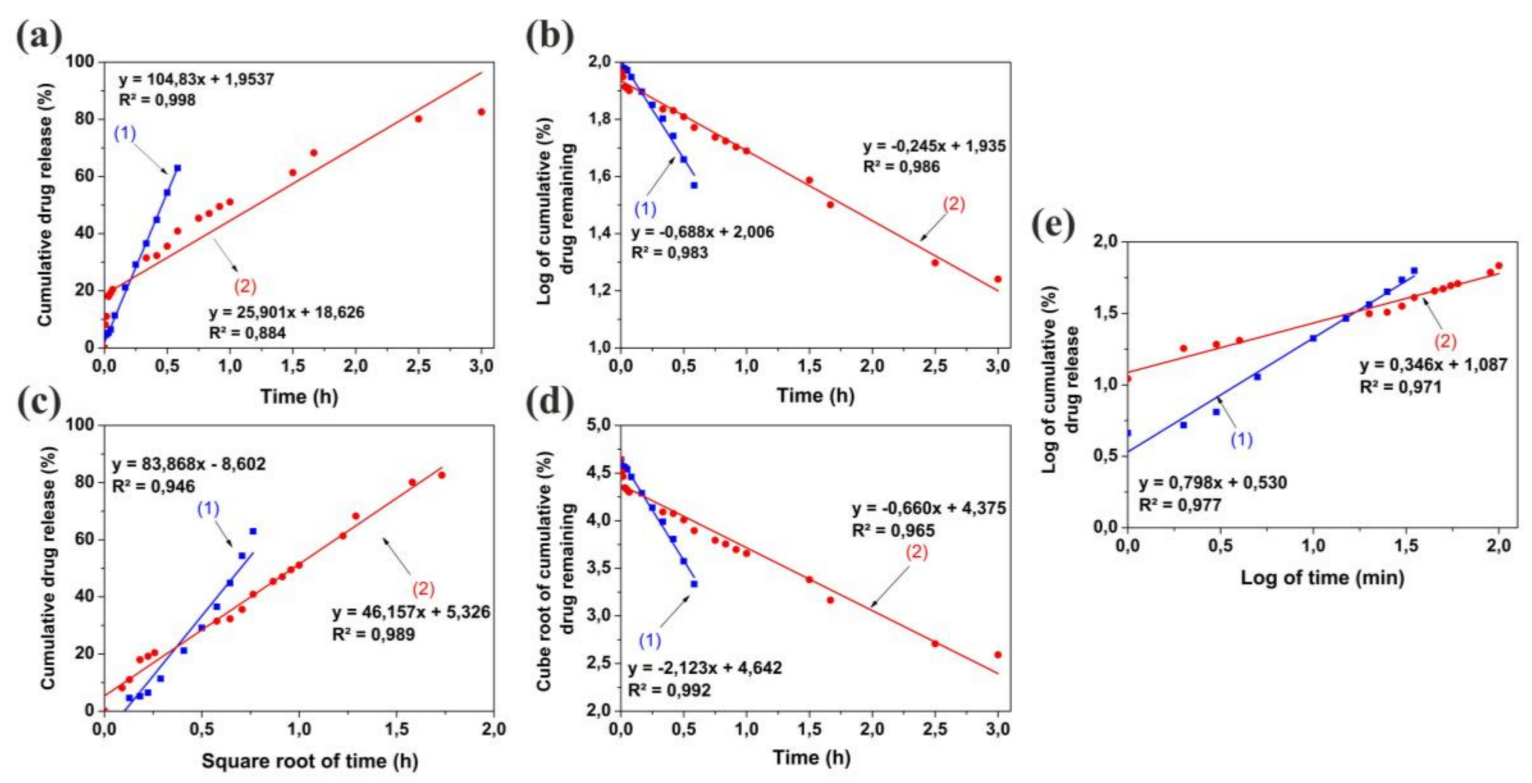
Kinetics study for the release of triclosan from CNC_TR(1) and CNC_TiO_2__TR(2): Zero order (**a**); First order (**b**); Higuchi (**c**); Hixon–Crowell (**d**) and Korsmeyer–Peppas (**e**) kinetic models.

The release mechanism of TR from the obtained samples was also investigated by using the Korsmeyer–Peppas, zero-order, first-order, Higuchi, and Hixon–Crowell kinetic models. Figure 8 displays the release of triclosan from CNC_TR (1) and CNC_TiO_2__TR (2) according to the various kinetic models. The CNC_TR sample exhibited zero order release kinetic profile with high R^2^ showing constant rate release behavior. The in vitro release profiles of the triclosan from CNC_TiO_2__TR could be best expressed by the Higuchi’s models, as the plots showed highest linearity (R^2^ from 0.99) indicating diffusion controlled drug release pattern (Figure 8b,c). Higuchi model is the most widely used model to describe drug release from an insoluble matrix, in which the release is governed by the diffusion of the drug through the matrix [54]. Thus, the release of triclosan from spherical cellulose nanocrystals as an insoluble matrix occurs by means of the kinetically controlled dissociation of the surface complexes combined with diffusion mechanisms. 

### 3.3. Antimicrobial Activity of the Obtained Nanocomposites

The purpose of the bacteria susceptibility test was to determine the retention of the antibacterial activity of the triclosan after release. For this, the antibacterial activity of TR released from the obtained nanocomposites films was investigated by the disk diffusion method against two bacterial strains, *S. aureus* and *E. coli*, representing a potential Gram-positive and Gram-negative pathogen, respectively. The results of the disk diffusion test are shown in Table 1 and Figure S1 (Supplementary). For CNC_TiO_2__TR and CNC_TR, inhibition zone diameters against *S. aureus* and *E. coli* were found to be 56 ± 2 mm and 38 ± 2 mm; 62 ± 4 mm and 42 ± 3 mm, respectively. The results confirmed that triclosan retains its medicinal properties after release from the nanocomposites. As can be seen from the Table 2, for Gram-negative *E. coli* the inhibition zone was smaller because this bacteria has a higher resistance than *S. aureus*, due to the specific composition of its cellular wall [55]. 

The inhibition zone of the nanocomposite obtained without using TiO_2_ as a binding agent (CNC_TR) against *E. coli* and *S. aureus* was observed higher than that for the CNC_TiO_2__TR sample. The possible explanation for this observation can be attributed to different release rates of triclosan from the nanocomposites. The enhanced speed of triclosan released from CNC_TR may result in increasing the antibacterial properties of the sample. 

It was also observed that the PCNC and CNC_TiO_2_ samples also demonstrated the antibacterial response against both strains. For PCNC, the diameters of the inhibition zone against *S. aureus* and *E. coli* were found to be 16 ± 7 mm and 13 ± 4 mm, respectively. This observation is in accordance with earlier studies, where the antibacterial effect of cellulose was obtained by its treatment with polycarboxylic acids [55,56,57]. For instance, Orhan and others [58] examined the antibacterial activity of the cotton fabrics treated with BTCA and citric acid against both *E. coli* and *S. aureus*. They found that the polycarboxylic acids were effective against both bacteria even at lower concentrations. The antibacterial efficiency of pure BTCA with concentration ranging from 7 to 350 μg/mL was confirmed against *Pseudomonas aeruginosa* and methicillin resistant *S. aureus* ATCC 33591 by agar well diffusion and micro broth dilution methods [59]. Pure nanocellulose does not show any antibacterial activity [60]. In case of the CNC_TiO_2_ sample, the diameters of the inhibition zone of *S. aureus* and *E. coli* were found to be 26 ± 6 mm and 19 ± 6 mm, respectively. Compared to PCNC, the increase of the diameter of the inhibition zone can be caused by the presence of TiO_2_ nanoparticles in the obtained nanocomposite. The neat crystalline titania nanoparticles (4 ± 1 nm) covered with an amorphous layer of triethanolamine used in the present study for the nanocellulose modification, are highly bio-digestible, biodegradable, non-toxic [61,62], and do not display any noticeable photochemical activity [43]. However, it was recently found that the presence of the carboxylic groups activates the crystalline core of titania and results in the appearance of its photocatalytic behavior. In the present work, no external stimuli like UV-irradiation were applied to activate titania during the bactericidal test. However, we suppose that the photocatalytic behavior of titania may result in the appearance of the bactericidal activity of the nanocomposite containing only grafted TiO_2_ nanoparticles (CNC_TiO_2_). 

## 4. Conclusions

In summary, we have successfully prepared nanocomposites films based on spherical-shaped cellulose nanocrystals and nano-titania with chemically immobilized model drug triclosan. The methodology developed in this study demonstrates that the spherical shaped cellulose nanocrystals could be produced directly from the filter paper as the model source with usage much lower concentrated acid hydrolysis (20 wt % of H_2_SO_4_) and shorter reaction time (1 h). The material is rather susceptible to soft scratching, even by a 2 M stift in a pencil test, but displays considerable tensile strength. The drug release studies showed long-term release profile of triclosan and can be described by Higuchi model. The results of bacterial susceptibility tests displayed that the released triclosan retained its antibacterial activity against *E. coli* and *S. aureus.* It was also found that a small amount of titania improved the antibacterial activity of the obtained nanocomposites, even without immobilization of model drug. 

## Figures and Tables

**Table 1:** Mechanical properties of the obtained spherical cellulose crystal-based films.

Sample	Tensile Strength (MPa)	Strain (%)	Young’s Modulus (MPa)
PCNC	1.3	4.4	3.7
CNC_TiO_2_	13.8	13.4	6.8
CNC_TiO_2__TR	11.0	12.6	6.3

**Table 2:** The measurements of antimicrobial activity of the obtained samples.

Sample	Type of Bacteria	MIC (mg/mL) [36]	Diameter of Inhibition Zone (mm ± SD ^1^)
PCNC	*E. coli* CCUG24T	-	13 ± 4
CNC_TiO_2_		-	19 ± 6
CNC_TR		0.1	42 ± 3
CNC_TR_TiO_2_		0.1	38 ± 2
PCNC	*S. aureus* CCUG1800T	-	16 ± 7
CNC_TiO_2_		-	26 ± 6
CNC_TR		0.1	62 ± 4
CNC_TR_TiO_2_		0.1	56 ± 2

(**^1^** SD—standard deviation).

## Supplementary Materials

The following are available online at http://www.mdpi.com/2079-4991/8/4/228/s1, the detailed explanation of the surface modification of cellulose nanocrystals; Table S1: IR-spectra of compound **1** and compound **2**; Figure S1: Results of inhibition zones of antibacterial activity against *E. coli* and *S. aureus*: PCNC (A,E), CNC_TiO_2_ (B,F), CNC_TiO_2__TR (C,G) and CNC_TR (D,H) samples.
