# Supplementary material for: Hybrid Drug Delivery Patches Based on Spherical Cellulose Nanocrystals and Colloid Titania—Synthesis and Antibacterial Properties
Source: Nanomaterials (Basel). 2018 Apr 8;8(4):228. doi: 10.3390/nano8040228 (PMC5923558; doi:10.3390/nano8040228)
Supplement: Supplementary file 1 [file nanomaterials-08-00228-s001.pdf]

nanomaterials

Supplementary Materials

Hybrid Drug Delivery Patches Based on Spherical Cellulose Nanocrystals and Colloid Titania — Synthesis and Antibacterial Properties

Olga L. Evdokimova [1,2], Fredric G. Svensson [2], Alexander V. Agafonov [1], Sebastian Håkansson [2], Gulaim A. Seisenbaeva [2] and Vadim G. Kessler [2,*]

[1] G.A. Krestov Institute of Solution Chemistry of the Russian Academy of Sciences, Akademicheskaya St.1, 153045 Ivanovo, Russia; olga_evdokimova@outlook.com (O.L.E.); ava@isc-ras.ru (A.V.A.)
[2] Department of Molecular Sciences, BioCenter, Swedish University of Agricultural Science, 750 07 Uppsala, Sweden; fredric.svensson@slu.se (F.G.S.); sebastian.hakansson@slu.se (S.H.); gulaim.seisenbaeva@slu.se (G.A.S.)
* Correspondence: vadim.kessler@slu.se; Tel.: +46-18-671541

Surface Modification of Cellulose Nanocrystals

Cellulose nanoparticles surface were modified by Triclosan in amounts calculated to achieve uniform, single layer coverage. The surface of titania was estimated using simple mathematical model where the particles are spherical, uniform and homogenous [1,2]. According to our assumption that drug bonded with the biopolymer through interaction with TiO_2, we firstly calculated the total number of titania nanoparticles, which was added to the nanocellulose, by following formula:

$$N_{total} = m_{total} / m_p$$

where m_{total} – total mass of TiO_2 particles (g), m_p – mass of the single TiO_2 particle (g).

The total surface area of titania nanoparticles grafted onto nanocellulose can be estimated as follows:

$$S_{total} = N_{total} \cdot S_p$$

where N_{total} – total number of titania nanoparticles, S_p - surface of the single TiO_2 particle (nm^2).

Then, the amount of drug n (mol) can be found as follows:

$$n = S_{total} \cdot N_A / S$$

where S – area of functional group (nm^2) and N_A is Avogadro number.

Table S1. IR-spectra of compound **1** and compound **2.**

Compound **1**	Compound **2**	Vibration
	687 s	*υ*(Ti-O-Ti)
805 s		*υ*(Ti-O)
816 s	815 s	*υ*(Ti-O-Ti)
894 s	894 s	*υ*(C-O-C)
	1488 m	*υ*(C=C) (arom)
1590 s	1593 m	*υ*(C=C) (arom)
1583 s	1582 s	*υ*(C=C) (arom)
1714 b	1712 m	*υ*(C=O)
3397 b		*υ*(-OH)

s = small, b = broad, m = medium.

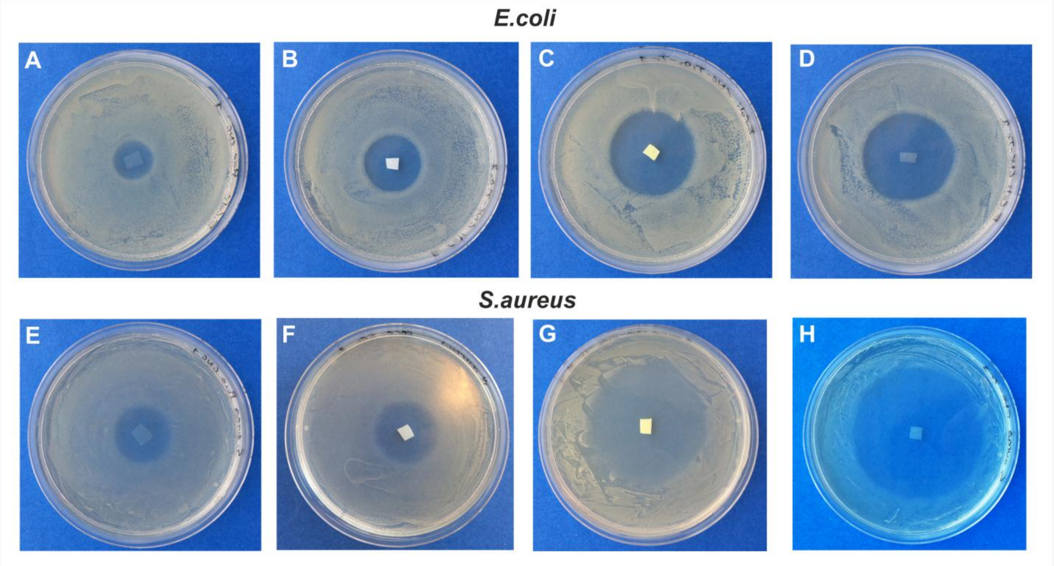

Figure S1 Results of inhibition zones of antibacterial activity against *E.coli* and *S.aureus*: PCNC (A,E), CNC_TiO_2 (B,F), CNC_TiO_2_TR (C,G) and CNC_TR (D,H) samples

References

1. Pazik, R.; Tekoriute, R.; Håkanson, S.; Seisenbaeva, G.A.; Wiglusz, R.; Strek, W.; Gun'ko, Y.K.; Kessler, V.G. Precursor and Solvent Effects in the Nonhydrolytic Synthesis of Complex Oxide Nanoparticles for Bioimaging Applications by the Ether Elimination (Bradley) Reaction. *Chem. Eur. J.* **2009**, *15*, 6820–6826.
2. Pazik, R.; Andersson, R.; Kepinski, L.; Nedelec, J.M.; Kessler, V.G.; Seisenbaeva, G.A. Surface Functionalization of the Metal Oxide Nanoparticles with Biologically Active Molecules Containing Phosphonate Moieties. Case Study of BaTiO3. *J. Phys. Chem. C* **2011**, 115, 9850–9860.
3. Galkina, O.L.; Ivanov, V.; Agafonov, A.V.; Seisenbaeva, G.A.; Kessler, V.G. Cellulose nanofiber–titania nanocomposites as potential drug delivery systems for dermal applications. *J. Mater. Chem. B* **2015**, *3*, 1688–1698.
